# Supplementary material for: Tailoring atomic layer growth at the liquid-metal interface
Source: Nat Commun. 2018 Nov 20;9:4889. doi: 10.1038/s41467-018-07381-w (PMC6244000; doi:10.1038/s41467-018-07381-w)
Supplement: Supplementary file 1 — Supplementary Information [file 41467_2018_7381_MOESM1_ESM.pdf]

Supplementary information for

Tailoring atomic layer growth at the liquid-metal interface

Cao et al

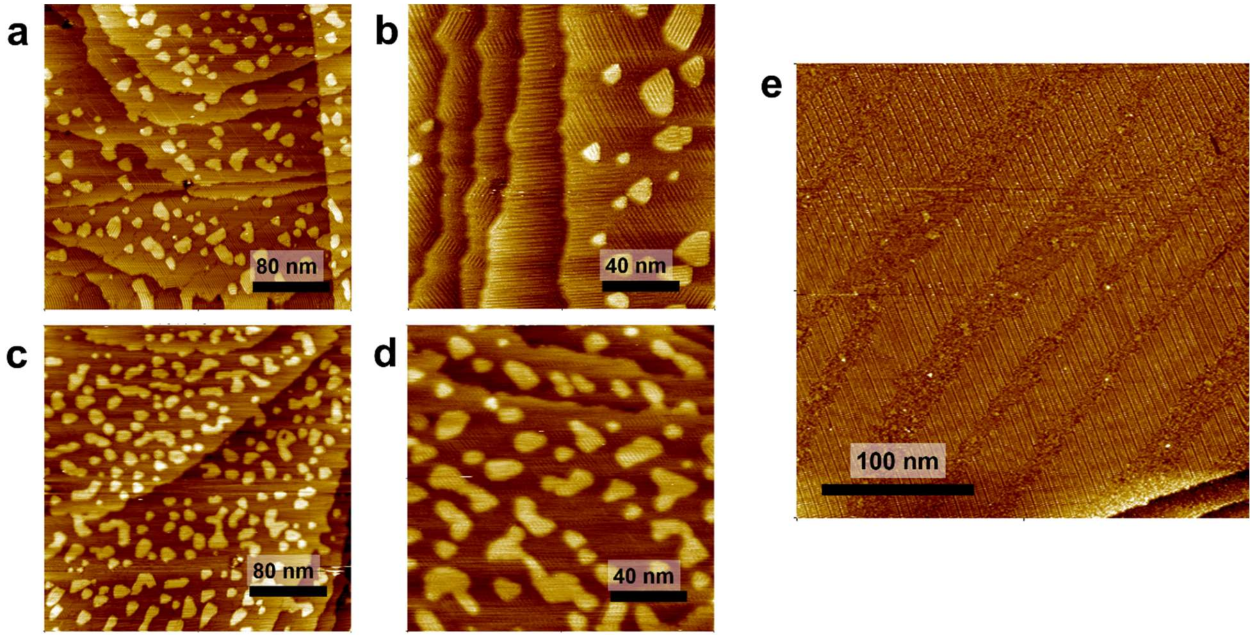

Supplementary Figure 1. Irregular Au islands and helicene superstructures. **a-d**, Supplementary large-scale STM images obtained by depositing TCB solutions of (*rac*)-Au_{38} on Au(111) surface at the concentration of 5 μM (**a**, **b**) and 25 μM (**c**, **d**), respectively. **e**, Supplementary large-scale STM image of the self-assembled structures of (*P*)-1 (C = 20 μM) at the TCB/Au(111) interface.

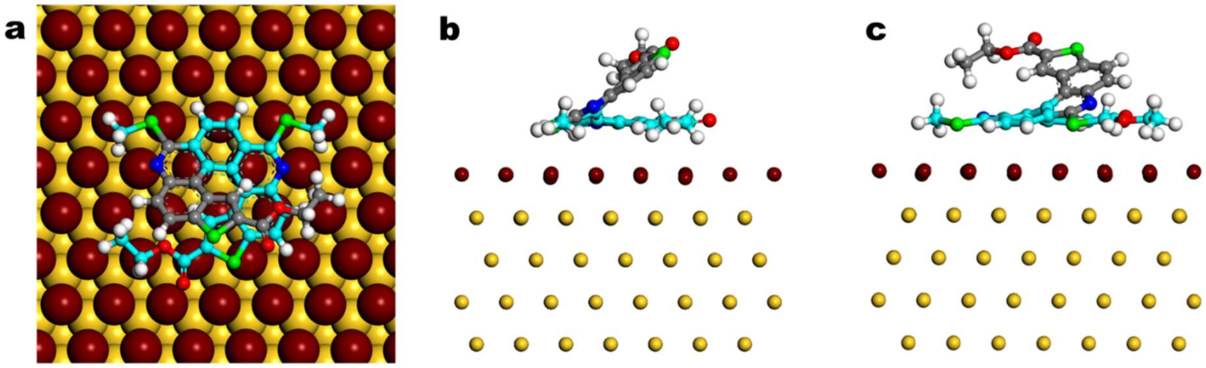

Supplementary Figure 2. Adsorption geometry of helicene on the Au(111) surface. **a**, Top, **b**, side and **c**, front views of the adsorption geometry of (*P*)-1 on Au(111) surface. The surface structure is described by a 7 × 7 supercell consisting of five monolayers of (111)-oriented Au slab and a vacuum layer of 20 Å. The adsorption geometry was first searched by a simulated quench method using the Compass II force field available in the Forcite module of Material Studio. A cutoff of 1.85 nm was applied for electrostatic and van der Waals interactions. 30 ps-long MD simulations are conducted in the NVT ensemble at 300 K with a time step of 1 fs. Quenching every100 steps yields 300 structures, of which the most stable structure was selected for further DFT calculations. The sulphur atoms of pendent thioether moieties appear atop surface Au atoms, in line with previous reports.[1-3]

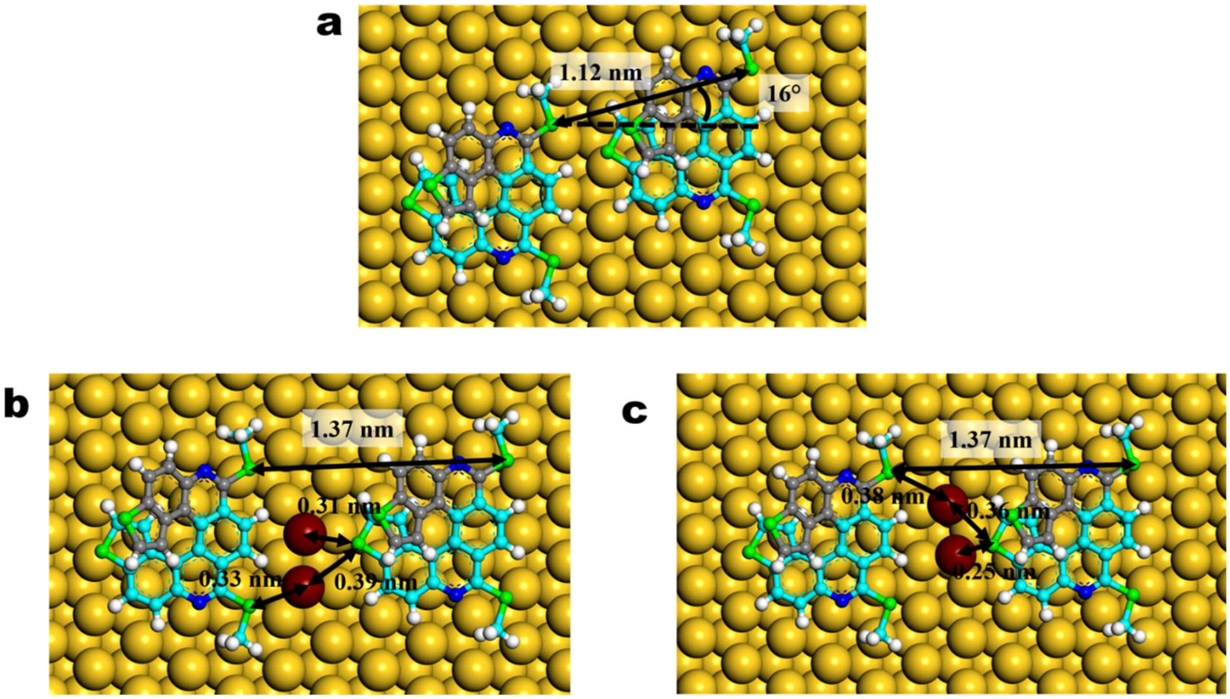

Supplementary Figure 3. Arrangements of helicenes in the absence and presence of Au adatoms. Adsorption geometries of **a**, a (*P*)-1 dimer and **b**,**c**, a Au-coordinated (*P*)-1 dimer on Au(111) surface. For the latter, two different situations were considered, revealing identical intermolecular distance. The surface is described by a 6 × 8√3 supercell consisting of three monolayers of (111)-oriented Au slab and a vacuum layer of 20 Å. To reduce calculation time, a simplified helicene structure and an energy cutoff of 300 eV were used. Geometry optimization started with the molecular arrangement that is observed is by STM. The bottom two Au layers are frozen in calculations while the surface layer, the Au adatoms and the helicene are fully relaxed until the convergence criteria reach 2×10^{-6} eV for SCF, 2×10^{-5} eV/atom for electronic structure, and 0.05 eV/Å for forces.

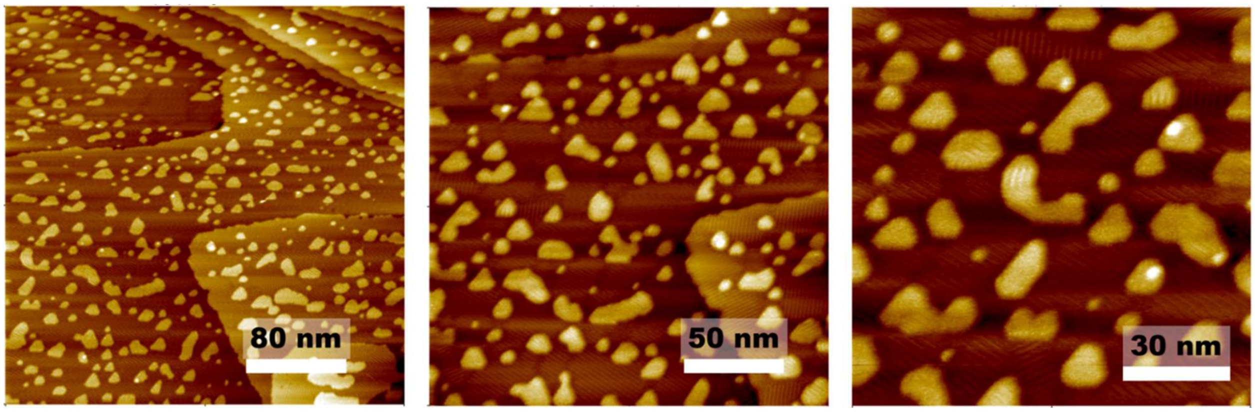

Supplementary Figure 4. **(*P*)-1:(*rac*)-Au_{38} = 2:5**. STM images acquired on Au(111) surface after the deposition of a premixed TCB solution containing 25 μM (*rac*)-Au_{38} and 10 μM (*P*)-1.

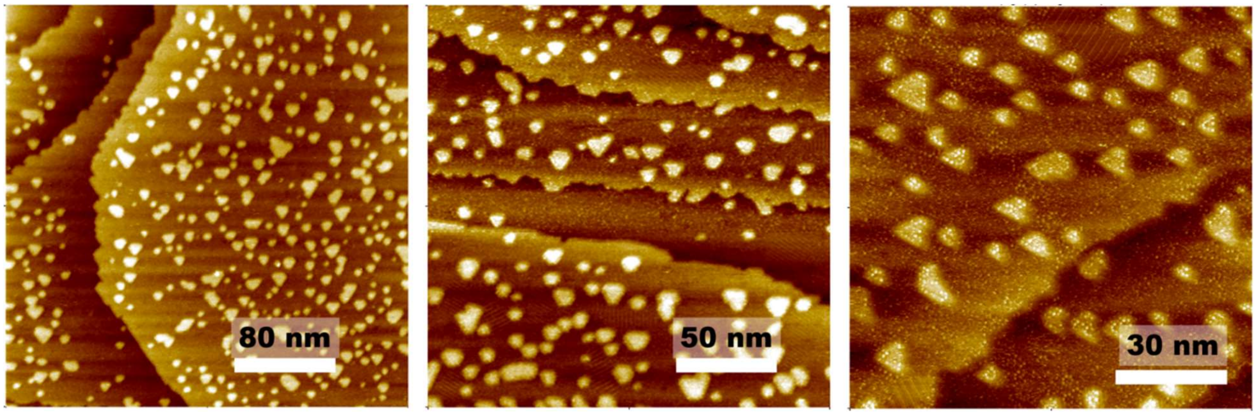

Supplementary Figure 5. (*P*)-1:(*rac*)-Au_{38} = 2:1. STM images acquired on Au(111) surface after the deposition of a premixed TCB solution containing 25 μM (*rac*)-Au_{38} and 50 μM (*P*)-1.

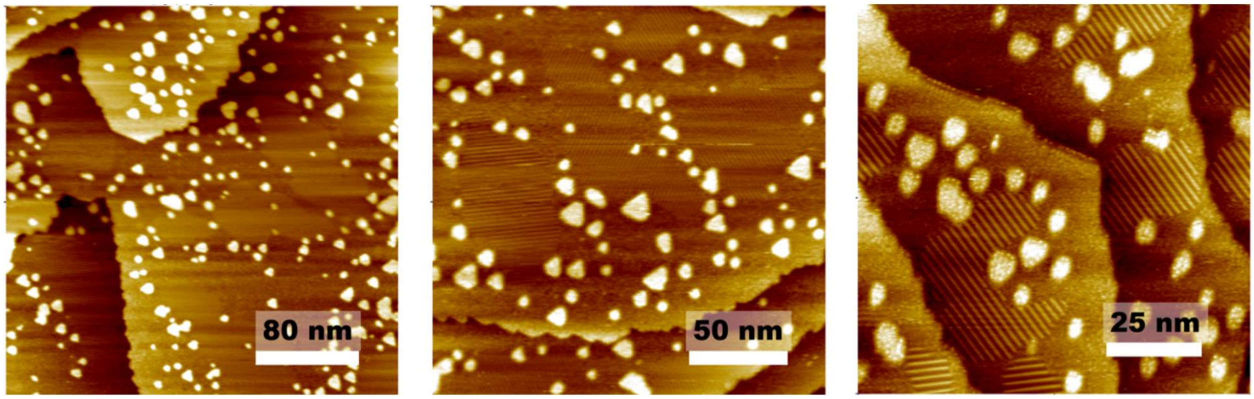

Supplementary Figure 6. (*P*)-1:(*rac*)-Au38 = 5:2. STM images acquired on Au(111) surface after the deposition of a premixed TCB solution containing 10 µM (rac)-Au38 and 25 µM (P)-1.

Supplementary Table 1. Statistics of the island properties upon depositing a TCB solution containing 25 µM (*rac*)-Au38.

Entry	Scanning area (nm^2)	Coverage of islands (%)	Number of islands	Average island size (nm^2)	Island density ($10^{-3}/nm^2$)
1	51597	30.0	74	209.2	1.43
2	38753	24.5	59	160.9	1.52
3	17792	23.4	32	130.1	1.80
4	17792	29.6	30	175.6	1.69
5	73948	25.4	125	150.3	1.69
6	111200	16.7	108	171.9	0.97
7	111200	26.0	88	328.5	0.79
8	27800	25.3	45	156.3	1.62

Supplementary Table 2. Statistics of the island properties upon depositing a premixed TCB solution containing 25 µM (*rac*)-Au_{38} and 10 µM (*P*)-1.

Entry	Scanning area (nm^2)	Coverage of islands (%)	Number of islands	Average island size (nm^2)	Island density ($10^{-3}/nm^2$)
1	37626	20.8	119	65.8	3.16
2	23700	17.4	35	117.8	1.48
3	148125	25.0	252	146.9	1.70
4	94800	21.4	191	106.2	2.02
5	23700	14.5	45	76.4	1.90
6	23700	18.8	40	111.4	1.69
7	94800	21.8	134	154.2	1.41

Supplementary Table 3. Statistics of the island properties upon depositing a premixed TCB solution containing 25 µM (*rac*)-Au_{38} and 25 µM (*P*)-1.

Entry	Scanning area (nm^2)	Coverage of islands (%)	Number of islands	Average island size (nm^2)	Island density ($10^{-3}/nm^2$)
1	21964	20.1	65	67.9	2.96
2	6034	18.2	26	42.2	4.31
3	21964	14.7	57	56.6	2.60
4	92800	14.2	150	87.9	1.62
5	88218	17.2	154	98.5	1.75
6	92800	18.3	173	98.2	1.86

Supplementary Table 4. Statistics of the island properties upon depositing a premixed TCB solution containing 25 µM (*rac*)-Au_{38} and 50 µM (*P*)-1.

Entry	Scanning area (nm^2)	Coverage of islands (%)	Number of islands	Average island size (nm^2)	Island density ($10^{-3}/nm^2$)
1	32832	20.5	139	48.4	4.23
2	22800	17.9	87	46.9	3.82
3	26594	12.7	107	31.6	4.02
4	63587	10.9	245	28.3	3.85
5	18263	24.2	75	58.9	4.11
6	30153	18.2	150	36.6	4.97

Supplementary Table 5. Statistics of the island coverage upon depositing a premixed TCB solution containing 25 µM helicene and 10-50 µM (*rac*)-Au_{38}.

	(*rac*)-Au38, 10 µM; helicene, 25 µM		(*rac*)-Au38, 25 µM; helicene, 25 µM		(*rac*)-Au38, 50 µM; helicene, 25 µM	
Entry	Scanning area (nm^2)	Coverage of islands (%)	Scanning area (nm^2)	Coverage of islands (%)	Scanning area (nm^2)	Coverage of islands (%)
1	21800	16.5	21964	20.1	33750	30.5
2	13150	14.4	6034	18.2	33750	30.3
3	21800	11.9	21964	14.7	15000	30.6
4	18320	13.4	92800	14.2	60000	27.3
5	21800	15.9	88218	17.2	15000	30.0
6	9690	19.7	92800	18.3	22326	25.7
7	60560	18.1	179300	27.0	21600	27.7
8	21800	17.5	92800	23.5	15000	26.5
9	9690	14.8	30680	21.0	33750	30.5
10			17480	18.6		

Note: Helicenes used in all the measurements are *P*-type, except for measurement 7-10 of the second sample, where *M*-type helicene was used.

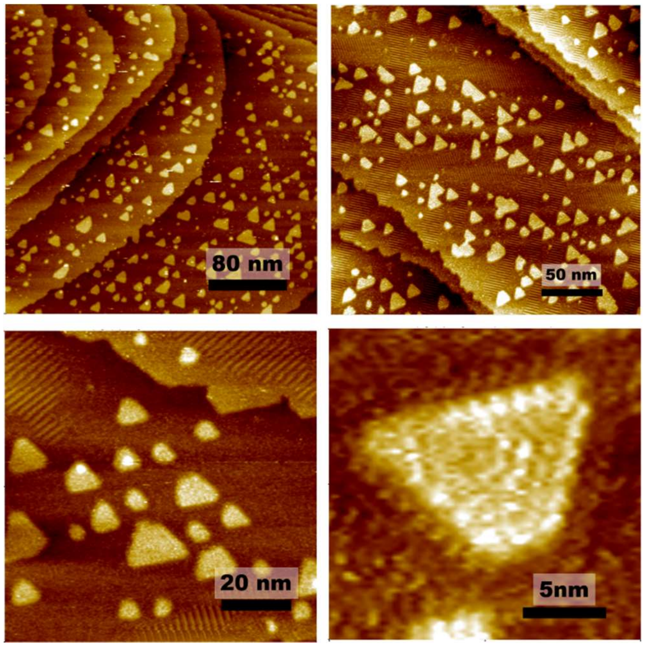

Supplementary Figure 7. (*M*)-1:(*rac*)-Au$_{38}$ = 1:1. STM images acquired on Au(111) surface after the deposition of a premixed TCB solution containing 25 µM (*rac*)-Au$_{38}$ and 25 µM (*M*)-1.

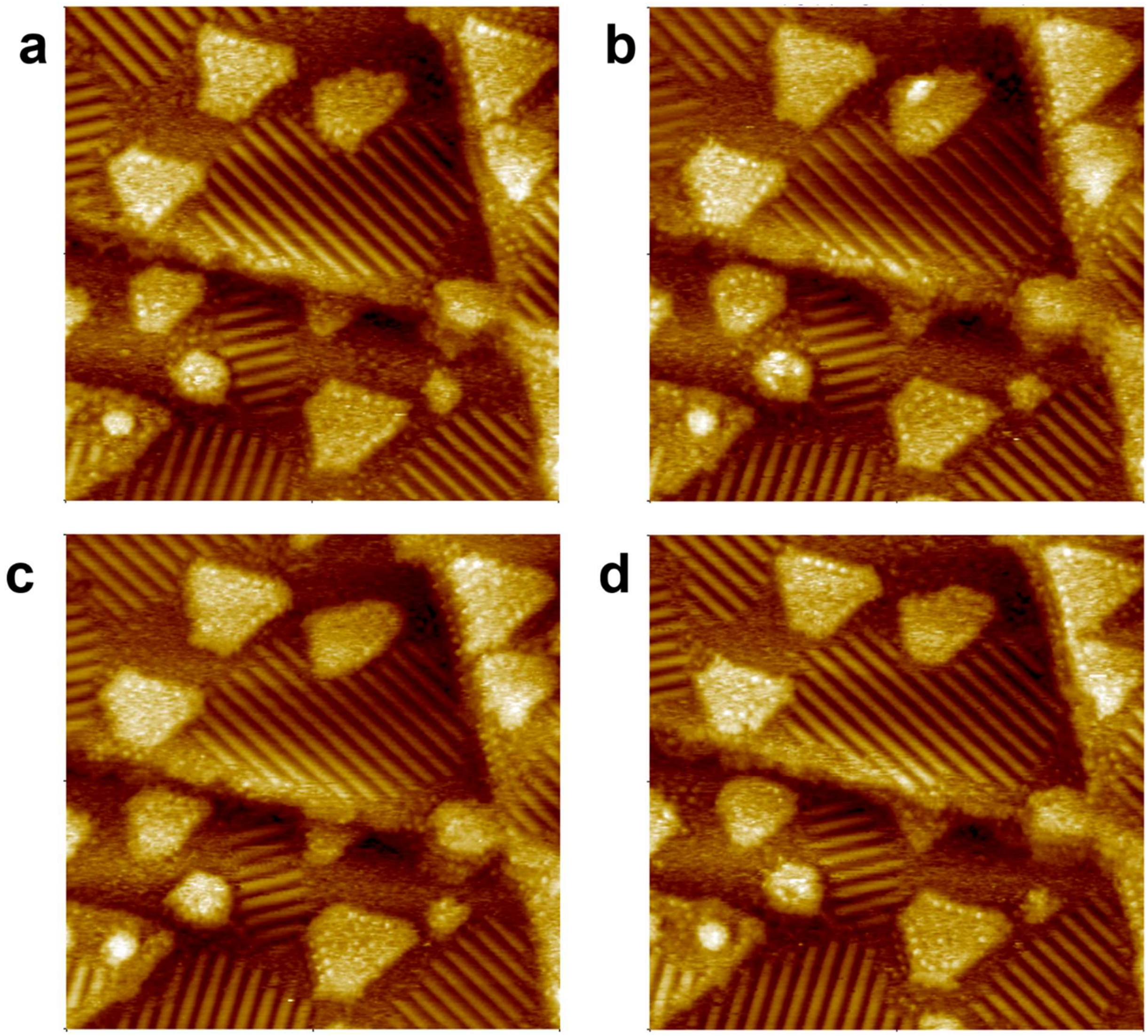

Supplementary Figure 8. Helicenes at the steps. Supplementary large-scale STM images (54 × 54 nm^2) showing the dynamic adsorption/desorption of helicenes at the steps and the formation of kink sites induced and stabilized by (*P*)-1. Close up images of **a**, **c** and **d** are presented in Fig. 5d-5f. However, at the location being examined, there is no significant variation to the island step between **a** and **b**.

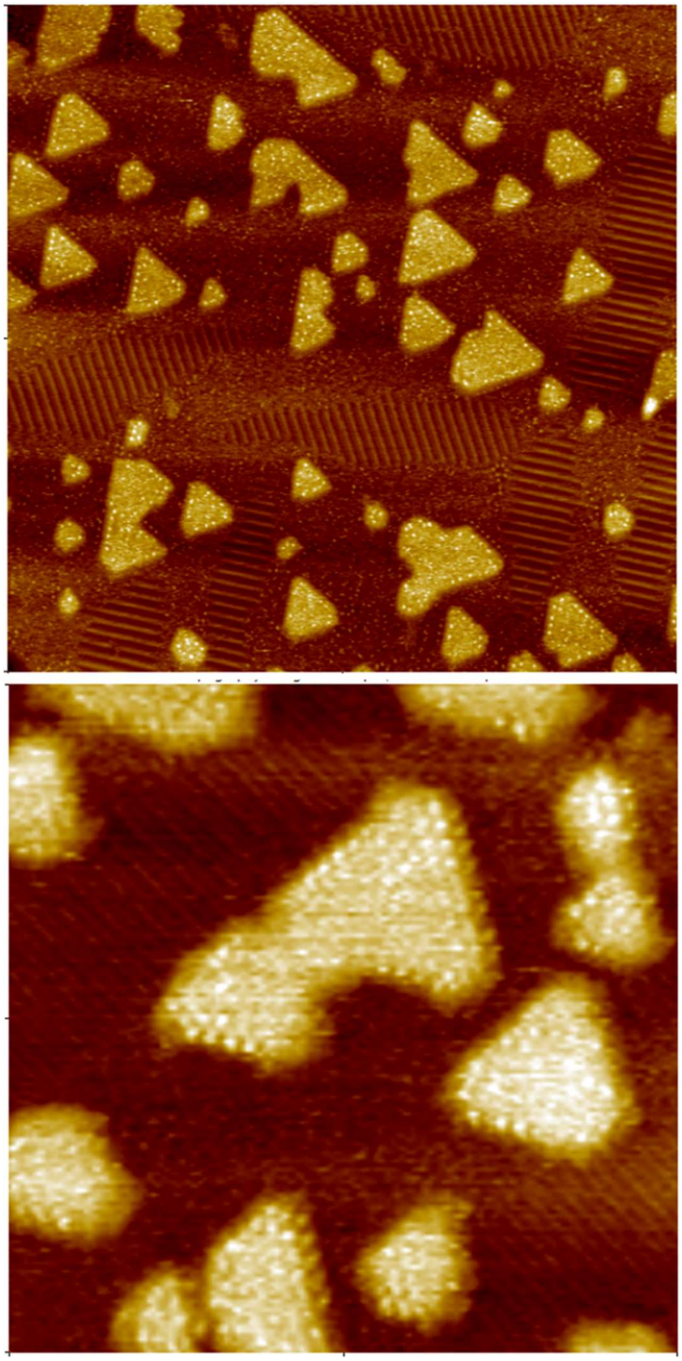

Supplementary Figure 9. Kink sites at the steps. Supplementary STM images showing the kink sites induced and stabilized by (*P*)-1. Top: 155 × 155 nm^2; bottom: 48 × 48 nm^2.

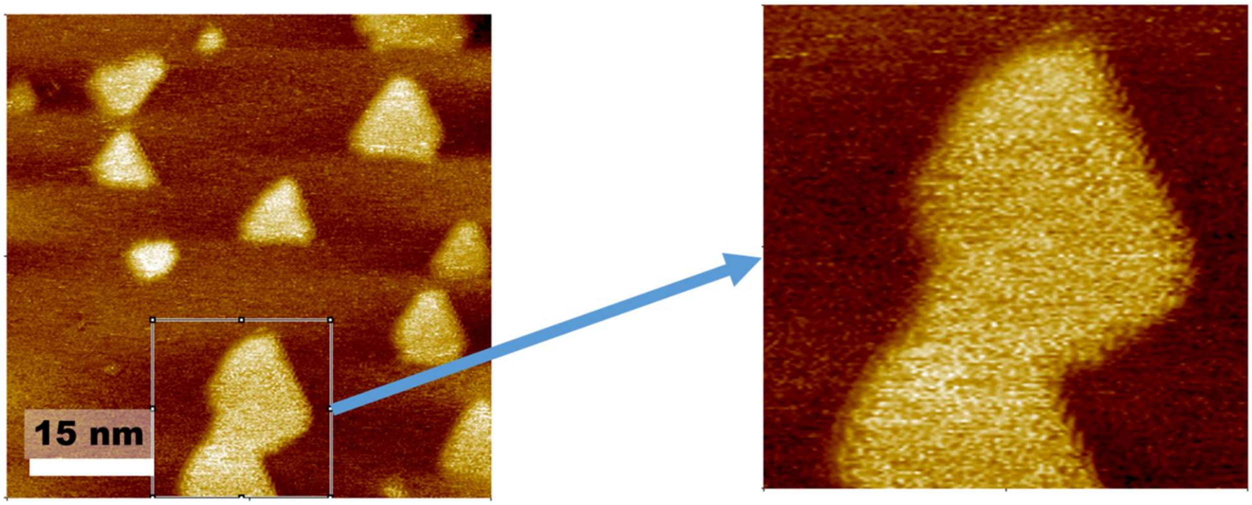

Supplementary Figure 10. Smaller kink sites. Supplementary STM images presenting the smaller kink sites after the removal, by the STM tip, of adsorbed helicenes.

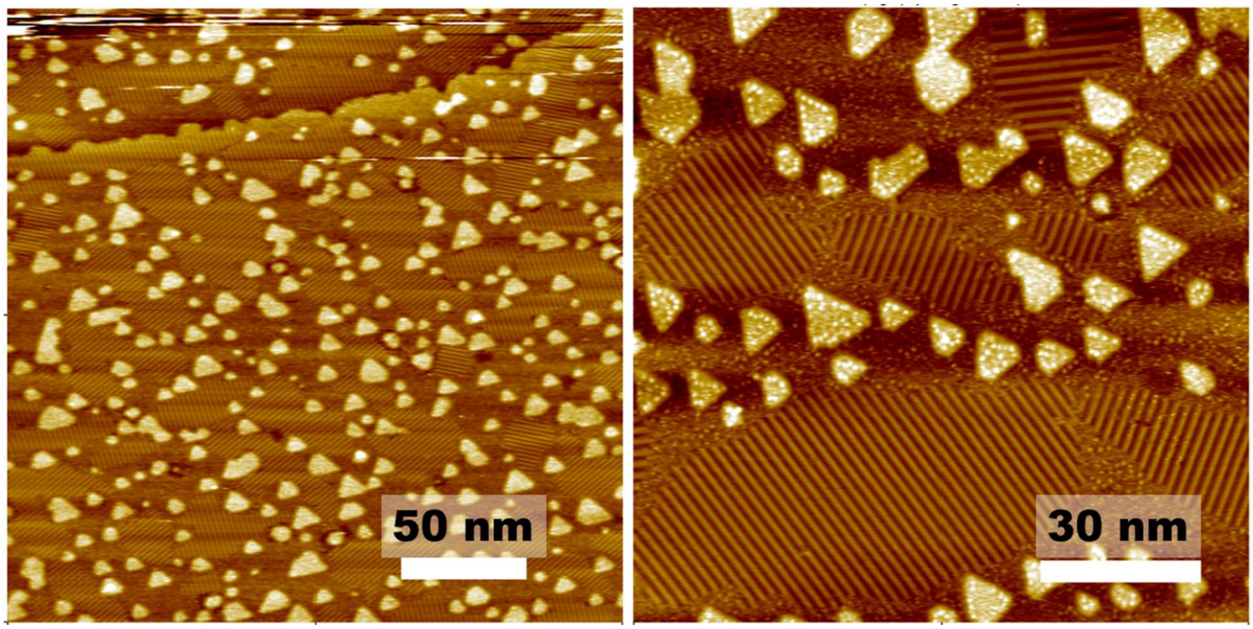

Supplementary Figure 11. (*rac*)-1 and (*rac*)-Au_{38}. STM images acquired on Au(111) surface after the deposition of a premixed TCB solution containing 25 µM (*rac*)-Au38 and 25 µM (*rac*)-1 (equimolar mixture of (*P*)-1 and (*M*)-1).

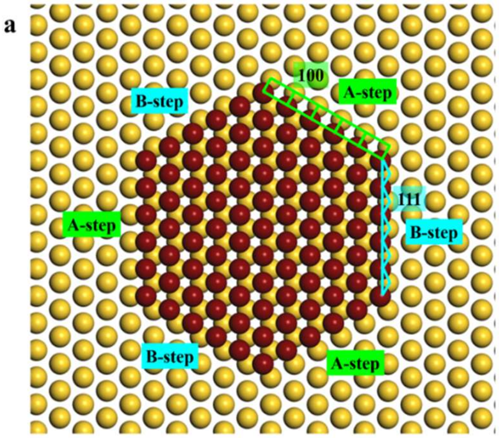

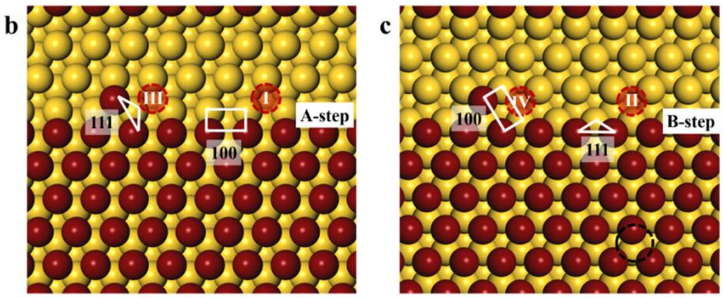

d

Binding energy (eV)

I	II	III	IV
3.632	3.658	3.795	3.782

Supplementary Figure 12. A-step *versus* B-step. **a**, The two types of close-packed steps along the $[1\bar{1}0]$ directions, (100)-faceted (A-type) and (111)-faceted (B-type). **b**, The difference between the attachment of a single Au atom and dimer formation at the A-step. A single Au atom (I) is attached to a A-microfacet, while a Au atom at III is facing A-microfacet in one direction but B-microfacet on the other one. **c**, The similar situation occurs to the B-step. **d**, As a consequence, the binding of a single Au atom to the B-step is slightly favored by 26 meV, but dimer and long string formation is more stable for the A-type. The binding energy difference between these two steps, however, is subtle and lower than kT at room temperature. $E_{bind} = -(E_{Au•step} - E_{Au} - E_{step})$.

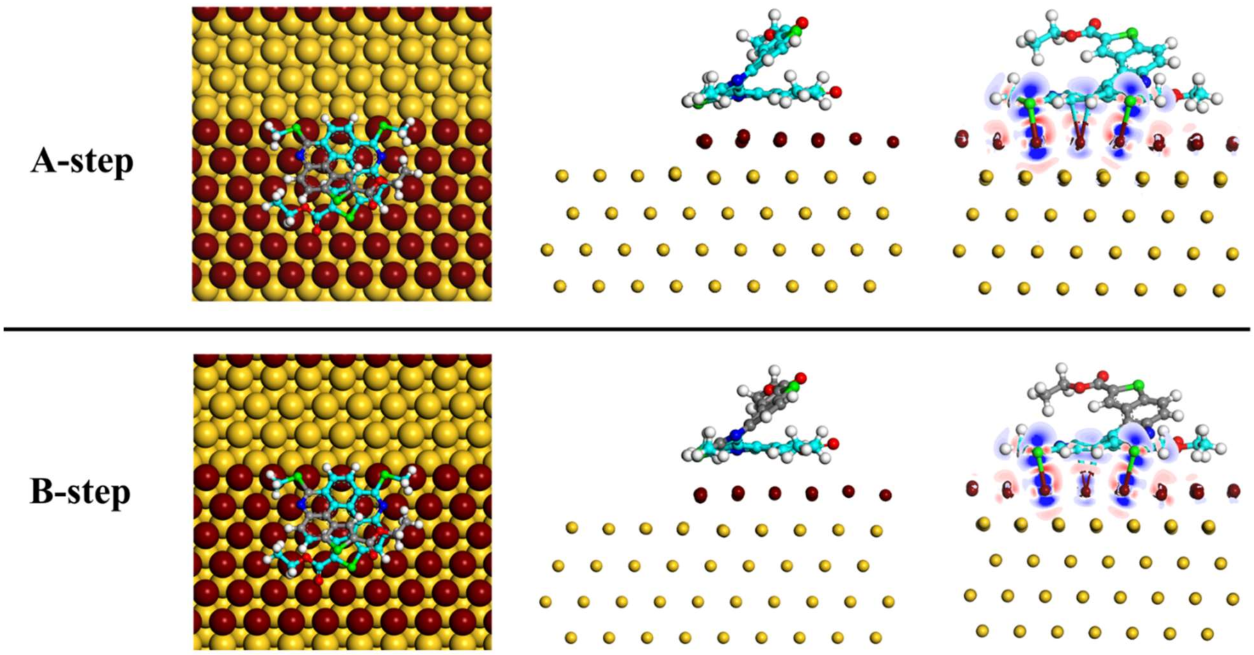

Supplementary Figure 13. Adsorption of helicene at the A-step and at the B-step. The optimized geometries of (*P*)-1 at two types of steps. In both cases the charge depletion and charge accumulation are displayed in blue and in red, respectively, with a contour value of 0.03 e $Å^{-3}$.

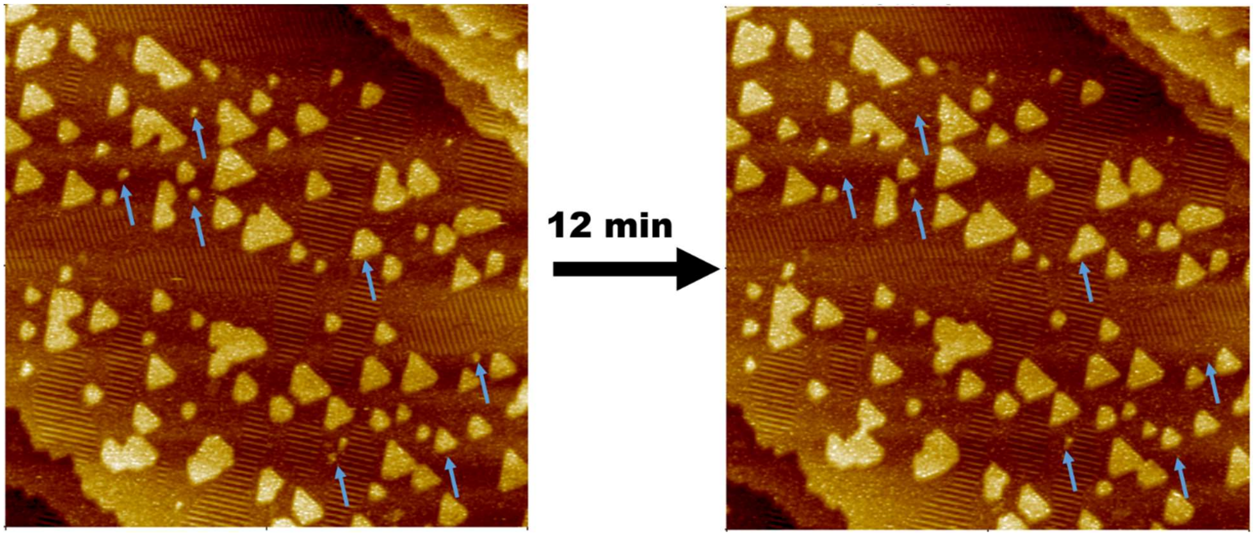

Supplementary Figure 14. Changes of islands over time. Sequential STM images recorded at the same place but in 12 minutes after the deposition of a premixed TCB solution containing 25 μM (*rac*)-Au38 and 25 μM helicene. The size of these images is 170 × 170 nm^2. The blue arrows indicate the changes.

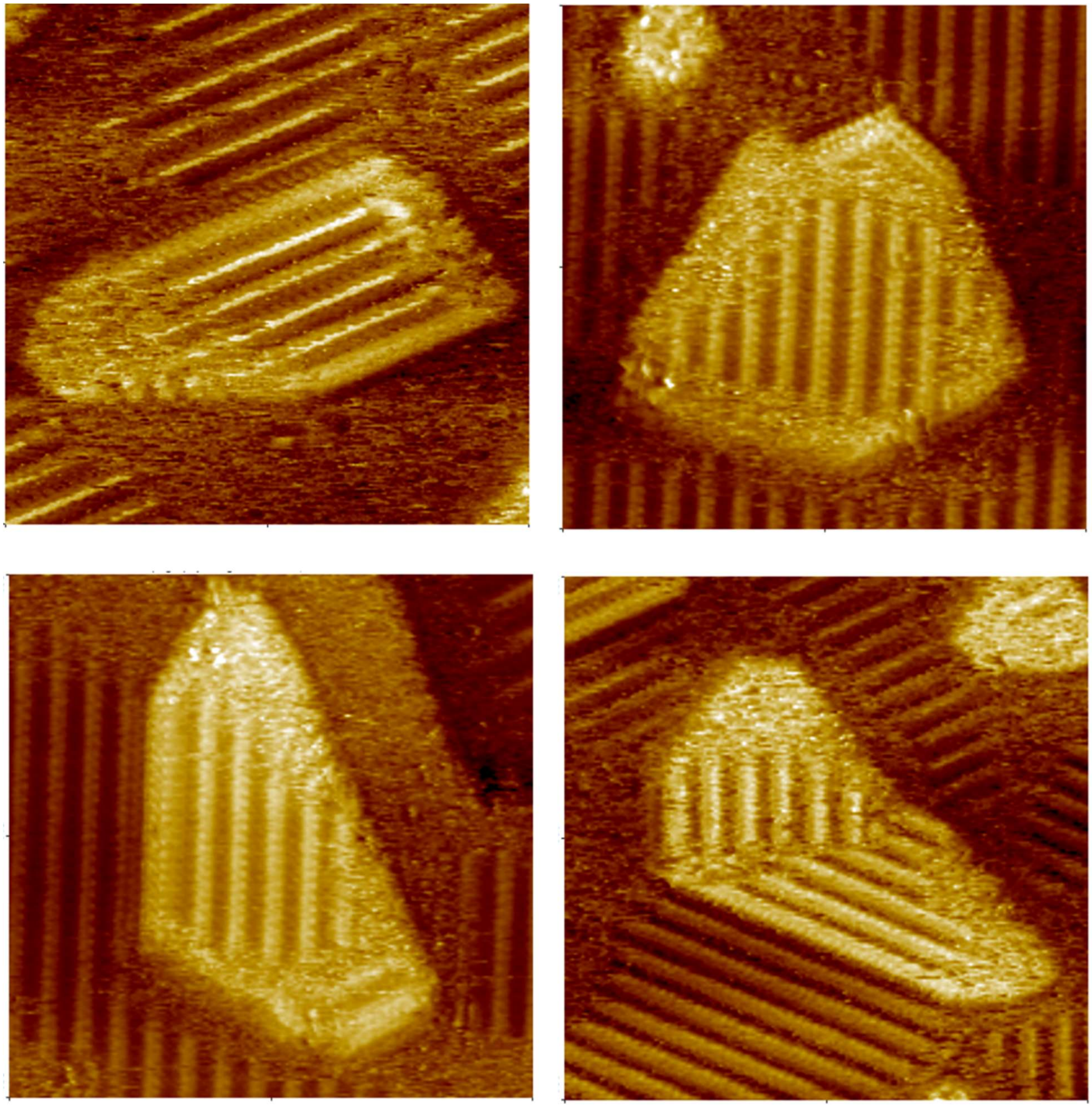

Supplementary Figure 15. Irregular islands decorated by PETs. STM images showing the irregular islands that are covered with PETs.

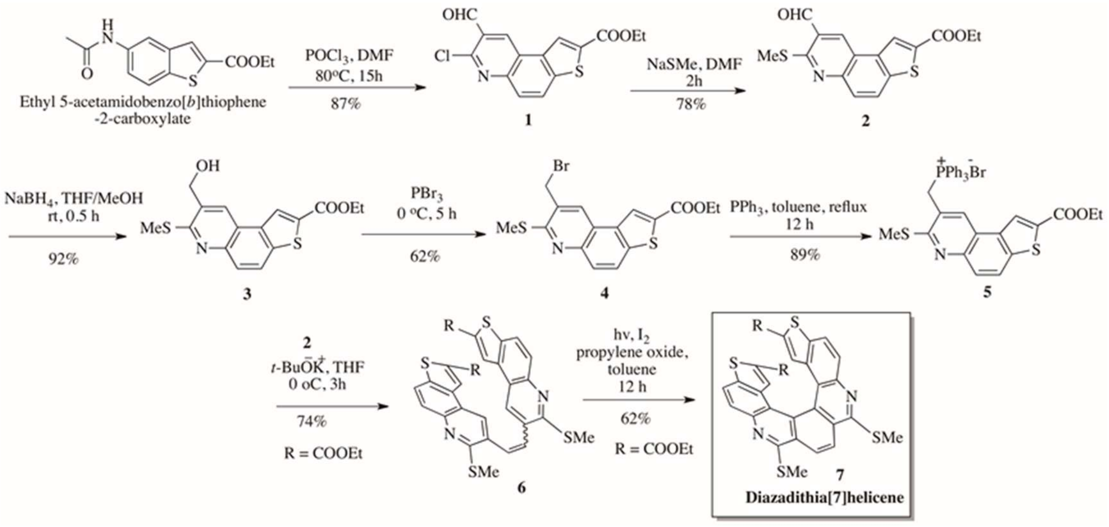

Supplementary Figure 16. Synthesis of (*P*)-1 and (*M*)-1. Schematic illustration of the synthesis of 7,10-bis(thiomethoxy)-6,11-diaza-3,14-dithia[7]helicene-2,15- bis(carboxylate). For clarity, compounds involved in the reactions are numbered **1** to **7**. Abbreviations: DMF, dimethylformamide; THF, tetrahydrofuran; MeOH, methanol.

Supplementary Note 1.

Ethyl 7-chloro-8-formylthieno [3,2-*f*]quinoline-2-carboxylate **1**, was used as the primary building block in this study, which was readily prepared from ethyl 5-acetamidobenzo[*b*]thiophene-2-carboxylate by Vilsmeier chloroformylation with DMF and $POCl_3$ at 80 °C for 15 h in 87% yield[4,5]. Ethyl 5-acetamidobenzo[*b*]thiophene-2-carboxylate was prepared from ethyl 5-aminobenzo[*b*]thiophene-2-carboxylate according to literature procedures[6]. Quinoline building block **1** was converted to ethyl 8-formyl-7-(methylthio)thieno [3,2- *f*]quinoline-2-carboxylate **2** by reaction with NaSMe in DMF at room temperature for 2 h in 78 % yield. Aldehyde **2** was converted to the corresponding alcohol **3** by reduction with $NaBH_4$ in THF/MeOH in 92% yield. This alcohol was converted to bromo derivative **4** by reaction with PBr_3 at 0 °C in THF, which was subsequently converted to the phosphonium salt **5** by reaction with triphenylphosphine in refluxing toluene for 12–15h in 89% yield. Wittig olefination of aldehyde **2** with phosphonium salt **5**, sodium *tert*-butoxide as base and THF as solvent at 0 °C for 3 h gave the corresponding symmetric azathiastilbene derivative **6** in 74 % yield. Derivative **6** was subjected to oxidative photocyclization[7] with iodine as oxidant and toluene as solvent to give

diazadithia[7]helicene **7** in 62% yield. Helicene **7** was completely characterized by nuclear magnetic resonance (NMR) spectroscopy and high-resolution mass spectrometry (HRMS)[8]. Chiral resolution: Enantiopure forms of diazadithia[7]helicene were obtained by HPLC separation on a ChiralPak IA chiral column at room temperature and a flow rate of 0.7 mL/min. The mobile phase was heptane/ethanol (60:40 v/v) under isocratic conditions.

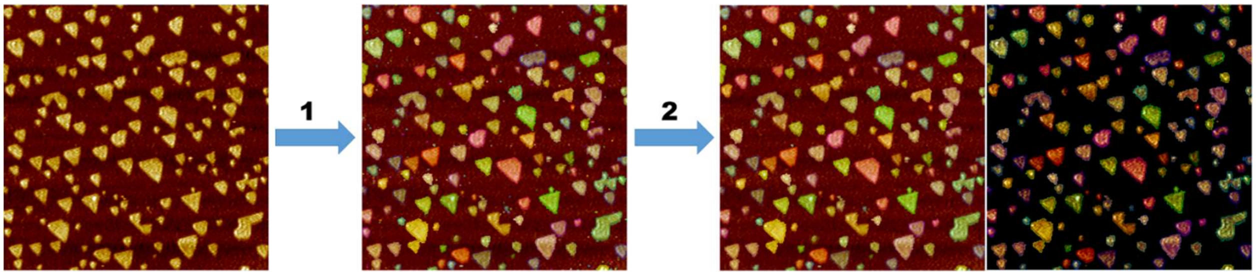

Supplementary Figure 17. Surface coverage analysis.

Supplementary Table 6. The calculated lattice constant of bulk Au, using PBE and PBE+D2 functional with different C6/R0 parameters for Au atom.

cutoff (eV)	*k*-mesh	PBE (Å)	PBE+D2 (Å)[a]	PBE+D2 (Å)[b]
300	6 × 6 × 6	4.199	4.131	4.165
	12 × 12 × 12	4.201	4.131	4.166
400	6 × 6 × 6	4.183	4.113	4.147
	12 × 12 × 12	4.184	4.114	4.149
600	6 × 6 × 6	4.185	4.115	4.149
	12 × 12 × 12	4.187	4.116	4.151
900	6 × 6 × 6	4.185	4.115	4.149
	12 × 12 × 12	4.187	4.116	4.151
Experiment		4.078		

[a]C6 = 422 eV Å^6, R0 = 1.772 Å (ref 9);
[b]C6 = 220 eV Å^6, R0 = 1.772 Å (ref 10).

Supplementary Table 7. The calculated cohesive energy of gold (eV/atom), using PBE and PBE+D2 functional with different C6/R0 parameters for Au atom (cutoff = 900 eV, k-mesh = 12 × 12 × 12).

**PBE**	**PBE+D2[a]**	**PBE+D2[b]**	**experiment[11]**
3.14	3.83	3.50	3.81

[a]C6 = 422 eV Å^6, R0 = 1.772 Å (ref 9);
[b]C6 = 220 eV Å^6, R0 = 1.772 Å (ref 10).

Supplementary References

1. Tierney, H. L., Calderon, C. E., Baber, A. E., Sykes, E. C. H. & Wang, F. Understanding the rotational mechanism of a single molecule: STM and DFT investigations of dimethyl sulfide molecular rotors on Au(111). *J. Phys. Chem. C* **114,** 3152–3155 (2010).
2. Tosoni, S., Boese, A. D. & Sauer, J. Interaction between gold atoms and thio-aryl ligands on the Au(111) surface. *J. Phys. Chem. C* **115,** 24871–24879 (2011).
3. Yang, B. et al. Stabilizing gold adatoms by thiophenyl derivatives: a possible route toward metal redispersion. *J. Am. Chem. Soc.* **134,** 11161–11167 (2012).
4. Meth-Cohn, O., Narine, B.& Tarnowski, B. A versatile new synthesis of quinolines and related fused pyridines. Part II. *Tetrahedron Lett.* **20,** 3111–3114 (1979).
5. Baruah, B. & Bhuyan, P. J. Synthesis of some complex pyrano[2,3-b]- and pyrido[2,3-b]quinolines from simple acetanilides via intramolecular domino hetero Diels–Alder reactions of 1-oxa-1,3-butadienes in aqueous medium. *Tetrahedron* **65,** 7099–7104 (2009).
6. Van Snick, W., Nulens, W., Jambons, S. & Dehaen, W. A facile synthetic route towards substituted thieno[3,2-e]indoles. *Synthesis* **5,** 767–774 (2009).
7. Waghray, D. et al. Synthesis and structural elucidation of diversely functionalized 5,10-diaza[5]helicenes. *J. Org. Chem.* **77,** 10176–10183 (2012).
8. Waghray, D. et al. Diazadithia[7]helicenes: synthetic exploration, solid-state structure, and properties. *Chem. Eur. J.* **19,** 12077–12085 (2013).
9. Amft, M., Lebègue, S., Eriksson, O. & Skorodumova, N. V. Adsorption of Cu, Ag, and Au atoms on graphene including van der Waals interactions. *J. Phys.: Condens. Matter*. **23,** 395001 (2011).
10. Tonigold, K. & Groß, A. Adsorption of small aromatic molecules on the (111) surfaces of noble metals: A density functional theory study with semiempirical corrections for dispersion effects. *J. Chem. Phys.* **132,** 224701 (2010).
11. Kittel, C. *Introduction to Solid State Physics*. John Wiley & Sons: New York, 2005.
